# The Theory and Practice of Surgery

**Published:** 1848

**Authors:** 


### ARTICLE I.

### McCLELLAN'S SURGERY.

*The Theory and Practice of Surgery.* By the late George McClellan, M.D. In one volume, 8vo. Edited by his son, J. H. B. McClellan, M.D. 1848. Grigg, Elliott, & Co., Phila.

We have received, from the publishers, just as this No. is going to press, an invoice of the above work, which has not yet arrived, but we deem it a duty we owe to the profession, to notify them without waiting until our next number, that a work so anxiously looked for by many, is already issued from the press. The great reputation of the author throughout the United States, cannot fail to be a sufficient guarantee of the value of the work. We will give a more extended notice in our next. (It will, in a few days, be for sale by Morrison & Talbott, Indianapolis.) E.

